# Pictorial essay: CT scan of appendicitis and its mimics causing right lower quadrant pain

**DOI:** 10.4103/0971-3026.37051

**Published:** 2008-02

**Authors:** Monika Sharma, Anjali Agrawal

**Affiliations:** Teleradiology Solutions, BA-49B, Phase-I, Ashok Vihar, New Delhi - 110 052, India

**Keywords:** Appendicitis, computed tomography

## Abstract

CT scanning is widely used in the diagnostic workup of right lower quadrant pain. While appendicitis remains the most frequent cause, a majority of patients referred for suspected appendicitis turn out to have alternative diagnoses or a normal CT study. The purpose of our pictorial essay is to present an overview of the CT findings of appendicitis and its common mimics and to highlight the features that provide clues to alternative diagnoses.

Right lower quadrant pain is a common clinical presentation. Because of low cost and easy availability, USG is sometimes preferred, but it is often inconclusive. Spiral CT is the imaging modality of choice in patients presenting with right lower quadrant pain as it helps make a definitive diagnosis in the majority of cases. The recognition of various pathologies on CT allows appropriate management and prevents unnecessary intervention in patients otherwise suspected to have appendicitis.

## Acute Appendicitis

**Figure 1(A, B):**
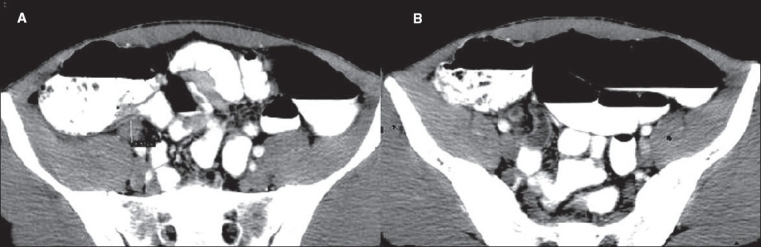
Appendicitis. Contrast-enhanced CT scan shows a thickened, fluid-filled tubular structure (arrow) in the right hemipelvis, contiguous with the cecal base

**Figure 2(A, B):**
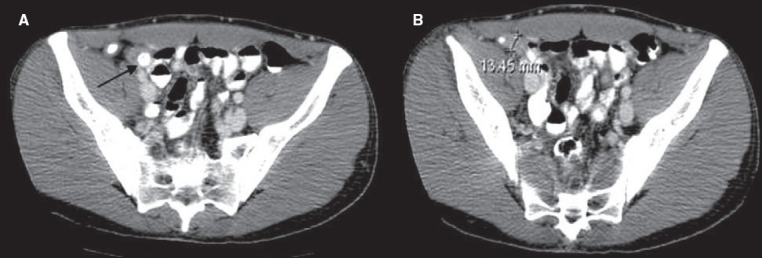
Appendicitis. Contrast-enhanced CT scan shows a dilated tubular structure in the right lower quadrant with appendicoliths (arrow)

**Figure 3:**
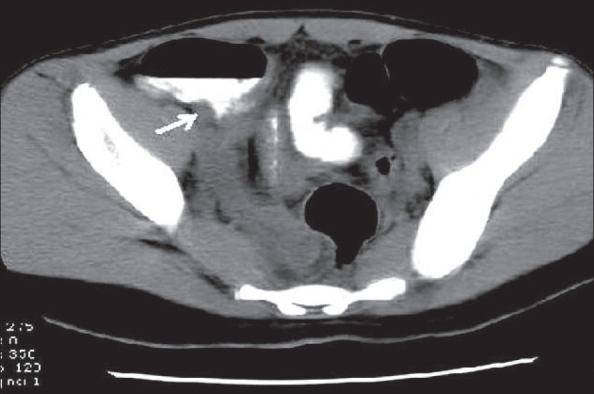
Arrowhead sign. Plain CT scan shows an arrowhead-shaped contrast collection in the cecum (arrow) due to extension of inflammation from the appendix to the cecum. The inflamed appendix is also seen

**Figure 4:**
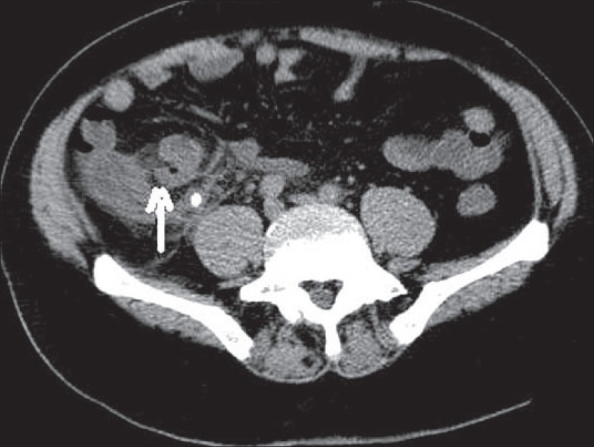
Perforated appendicitis. Plain CT scan shows a dilated appendix with an appendicolith (arrowhead) and periappendiceal abscess formation (arrow)

The CT diagnosis of acute appendicitis is based on the presence of a dilated, thick-walled, blind-ending, tubular structure with a diameter exceeding 6 mm, periappendiceal inflammation, and mucosal hyperenhancement, with or without an appendicolith[1,2] [Figures 1 and 2]. There may be thickening of the cecal base or terminal ileum due to contiguous inflammation. Focal thickening of the cecal apex results in an arrowhead-shaped contrast collection in the cecum, which points to the occluded appendiceal lumen (arrowhead sign), a secondary sign of acute appendicitis [Figure 3].[3] Enlarged mesenteric lymph nodes may be seen in the right lower quadrant. Discontinuous wall enhancement or a focal defect in the wall of the inflamed appendix, suggest perforation. Extraluminal air loculi or a loculated fluid collection/abscess may be seen in cases of frank appendiceal perforation [Figure 4].

**Figure 5:**
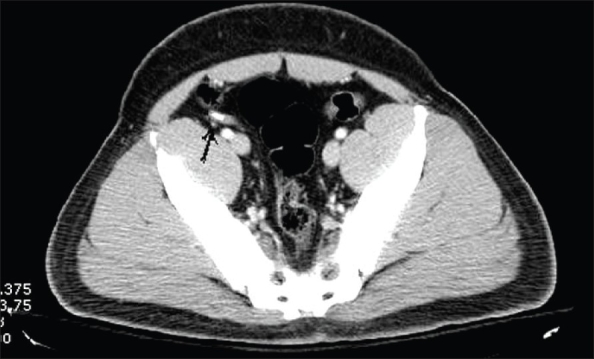
Appendicolith without appendicitis. Contrast-enhanced CT scan shows a nonobstructive appendicolith (arrow) in a normal-caliber appendix without periappendiceal inflammation

The diagnosis may be difficult in mild or incipient forms of appendicitis, in which there may be borderline enlargement of the appendix with subtle wall enhancement but without periappendiceal inflammation. There is considerable overlap between the maximum diameters of normal and abnormal appendices. A 6- to 8-mm appendix may be normal even if it is fluid-filled and a 9-mm appendix may also be normal in the absence of periappendiceal fat stranding. Some investigators consider a 12-mm diameter appendix as definitely abnormal,[4] if the appendix is less than 9 mm in an asymptomatic patient, the likelihood of appendicitis is considered very small.[4] The presence of a calcified appendicolith, without periappendiceal inflammatory changes, also does not necessarily indicate acute appendicitis [Figure 5].[5] Appendicitis can be excluded on a good quality CT scan in a patient with adequate intraabdominal fat even if the appendix itself cannot be seen, provided secondary CT signs of appendicitis are absent.[6]

**Figure 6(A, B):**
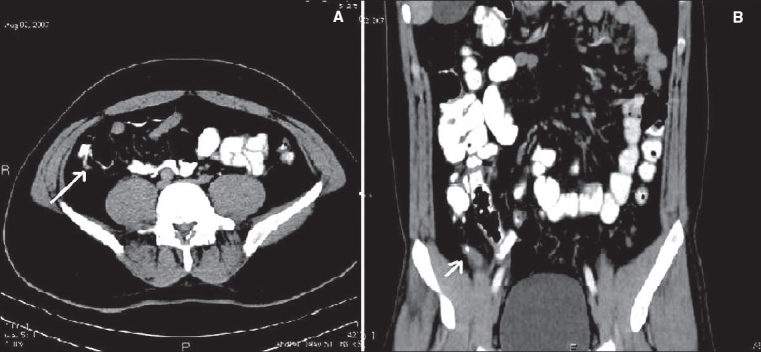
Distal appendicitis. Contrast-enhanced axial CT scan (A) shows that the proximal appendix is contrast-filled (long arrow). The coronal reformatted image (B) shows focal periappendiceal inflammatory changes and a thickened distal appendix (short arrow)

## Factors Contributing to a False Negative Diagnosis of Acute Appendicitis

**Figure 7:**
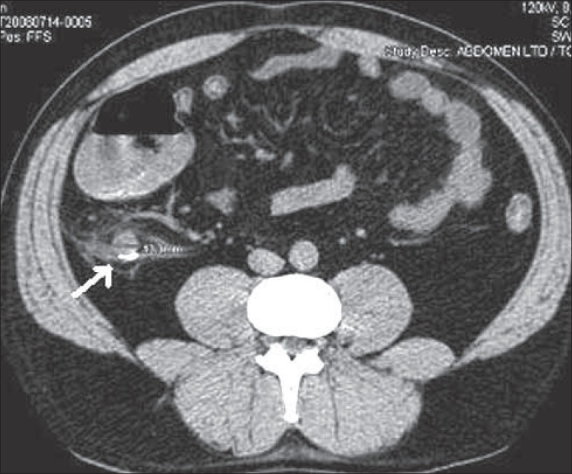
Stump appendicitis. This patient is status post-appendicectomy. Plain CT scan shows inflammatory changes in the right lower quadrant with a dilated tubular structure (arrow)

- **Anatomic alterations in the location of the appendix:** Identification of the appendix may be difficult because of its variable position and length. Evaluation of the anatomy of the cecum and ileocecal valve helps in locating the position of the normal appendix. The appendix is most frequently retrocecal in position. In cases of bowel malrotation, the cecum and appendix may be located to the left of the midline.
- **Distal appendicitis:** This can be a diagnostic challenge for radiologists. Sometimes, the proximal appendix is air-filled and the distal portion is fluid-filled and dilated with focal periappendiceal inflammatory changes [Figure 6]. Cecal base thickening will be absent in such cases. Therefore, it is necessary to completely trace the appendix from the cecal base to its distal-most portion to make a correct diagnosis.
- **Nonopacification of the cecum and distal ileum:** Complete opacification of the cecum and distal ileum helps in identifying the exact location of the normal or abnormal appendix. In the absence of oral contrast, a fluid-filled, normal small bowel loop may mimic an enlarged appendix.
- **Paucity of intraabdominal fat:** This represents the most common reason for a false negative diagnosis of acute appendicitis.[7] Usually, in children and patients with lean body habitus, there is relative paucity of intraabdominal fat, which may result in nonvisualization of the appendix or of the periappendiceal inflammatory changes. In patients with less body fat, use of oral/rectal contrast is important. The small bowel opacified with oral contrast may help identify a nonopacified distended appendix.
- **Small bowel dilatation or abscess formation:** In acute appendicitis, there may be small bowel dilatation in the right lower quadrant, which may mimic small bowel obstruction. Therefore, if there is small bowel dilatation in the right lower quadrant without a definite cause of obstruction, the diagnosis of acute appendicitis should be considered.[8] Ruptured appendicitis should be suspected in the presence of extensive inflammatory changes with phlegmon and abscess formation, despite a nonvisualized appendix.
- **Stump appendicitis:** Acute inflammation of the appendiceal stump is a rare complication of appendicectomy. With the increasing use of laparoscopic appendicectomy, there is an increase in the number of cases of stump appendicitis. A residual stump of greater than 5 cm increases the chances of stump appendicitis.[9] CT features include pericecal inflammation with fat stranding, adjacent to the appendiceal stump [Figure 7]. In view of this, it is important to understand that a past history of appendicectomy does not necessarily exclude the diagnosis of appendicitis.

## Common Differential Diagnoses

### 1. Inflammatory bowel disease

**Figure 8:**
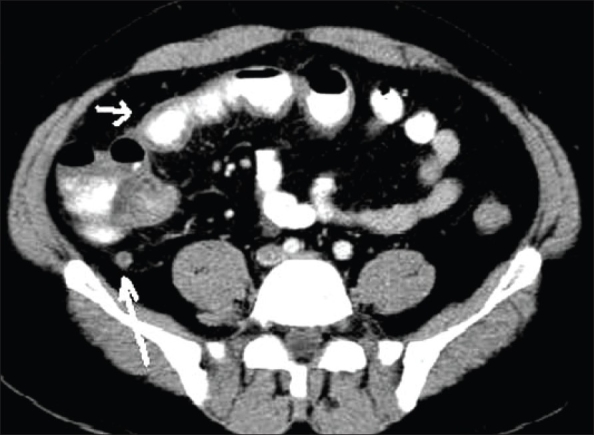
Crohn's disease. Contrast-enhanced CT scan shows distal ileal loops with long segment circumferential wall thickening (short arrow) and adjacent fat stranding and skip lesions. The normal appendix is also seen (long arrow)

Crohn's disease is an inflammatory disorder of unknown etiology, presenting most commonly in the second and third decades of life. It commonly involves the terminal ileum and can clinically mimic appendicitis. CT findings include bowel wall thickening, increased attenuation of mesenteric fat, skip lesions, mesenteric fibrofatty proliferation (creeping fat), and mesenteric lymphadenopathy [Figure 8].[1] Abscess and fistula formation are known complications. The visualization of a normal appendix, the presence of the epicenter of inflammation away from the appendix, with predominant pericecal inflammatory changes and terminal ileal wall thickening are findings that favor a diagnosis of inflammatory bowel disease.

### 2. Right ureteric obstruction

**Figure 9:**
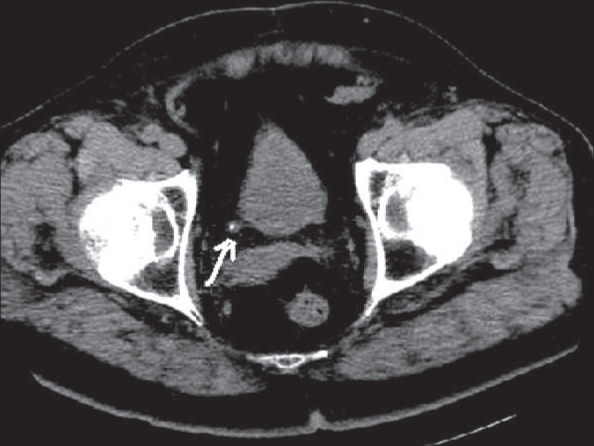
Ureteric calculus. Plain CT scan shows a ‘soft tissue rim’ sign caused by edema of the ureteric wall surrounding a calculus at the site of impaction (arrow)

**Figure 10(A, B):**
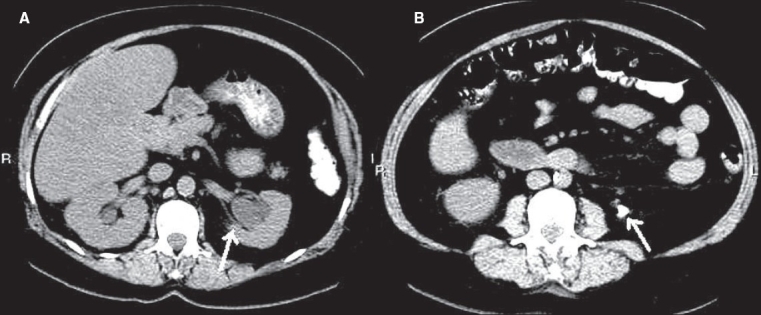
Ureteric calculus. Plain CT scan shows left hypdronephrosis (arrow in A) due to a 6 mm calculus (arrow in B). Note the absence of the ‘soft tissue rim’ sign

This commonly presents with right flank or lower quadrant pain. Ureteric or collecting system dilatation and direct visualization of the stone at the level of obstruction help clinch the diagnosis of ureteric calculus. The ‘soft tissue rim’ sign, i.e., the presence of soft tissue density around the ureteric calculus at the site of obstruction, which is secondary to ureteric wall edema [Figure 9], is helpful in differentiating a ureteric calculus from a phlebolith. However, this sign may be absent if the stone is impacted at the ureterovesical junction or if the stone size is more than 4 mm[10] [Figure 10A, B]. The other secondary signs of ureteric obstruction include perinephric fat stranding, renal enlargement,[11] and reduced attenuation by more than 5 HU as compared to the nonobstructed kidney.[12] This difference in attenuation is related to edema in the obstructed kidney (pale kidney sign).[12]

**Figure 11:**
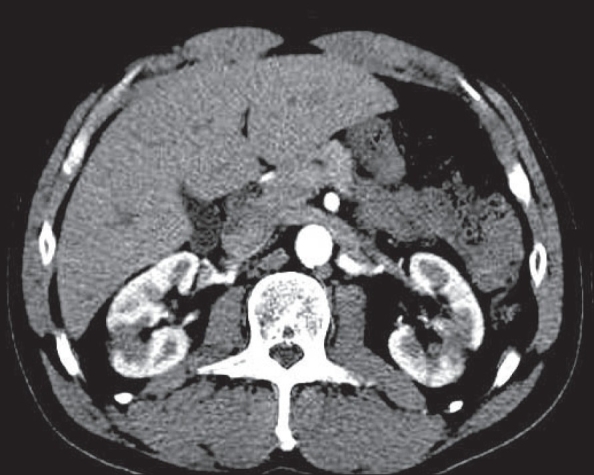
Acute pyelonephritis. Contrast-enhanced CT scan shows enlarged bilateral kidneys with streaky hypodensities (arrows) and minimal perinephric fat stranding

**Figure 12(A, B):**
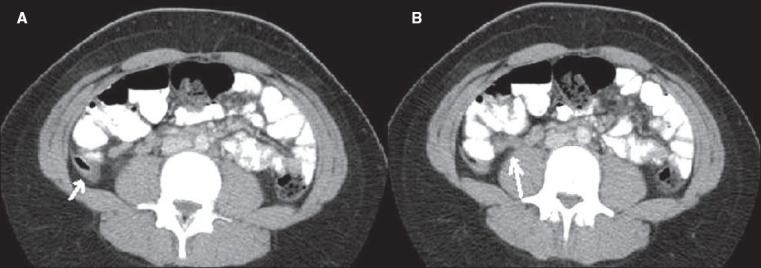
Mesenteric adenitis. Contrast-enhanced CT scan shows enlarged mesenteric lymph nodes (long arrow in A) in the right lower quadrant mesentery. The appendix is normal (short arrow in B)

### 3. Acute pyelonephritis

Acute pyelonephritis can present with right flank pain or lower abdominopelvic pain. Noncontrast CT may demonstrate normal or enlarged kidneys. Perinephric fat stranding and thickening of the renal fascia are seen. Occasionally, on unenhanced CT, high attenuation areas may be seen, suggesting hemorrhage. On contrast-enhanced CT, wedge-shaped areas of decreased parenchymal enhancement, with focal or diffuse renal enlargement, are noted [Figure 11]. A striated pattern of alternating linear increased and decreased attenuation in the kidney (striated nephrogram) may also be seen.

### 4. Mesenteric adenitis

This is a benign infection or inflammation of lymph nodes within the mesentery. Its clinical presentation mimics appendicitis. Diagnostic criteria are enlarged mesenteric lymph nodes in the right lower quadrant (short axis diameter >5 mm; 3 or more in number) with or without associated ileal or ileocecal wall thickening, in the setting of a normal appendix[13] [Figure 12].

### 5. Ileocecal tuberculosis

**Figure 13(A, B):**
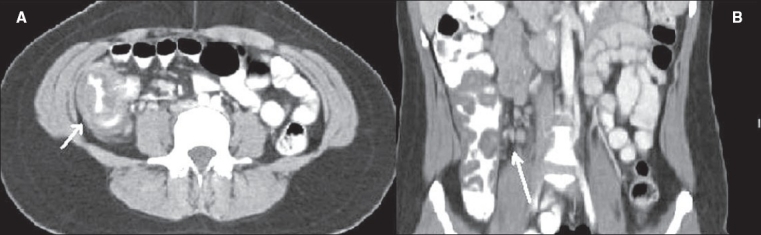
Ileocecal tuberculosis. Contrast-enhanced axial (A) and coronal (B) CT scan images show circumferential wall thickening of the cecum and terminal ileum (arrow in A) and enlarged mesenteric lymph nodes (arrow in B)

The ileocecal valve, adjacent terminal ileum, and cecum are involved in 90% of gastrointestinal tuberculosis.[14] CT findings include circumferential wall thickening of the cecum, terminal ileum, and ileocecal valve, with associated enlarged mesenteric lymph nodes [Figure 13]. The lymph nodes usually demonstrate central necrosis due to caseation.

### 6. Epiploic appendagitis

**Figure 14:**
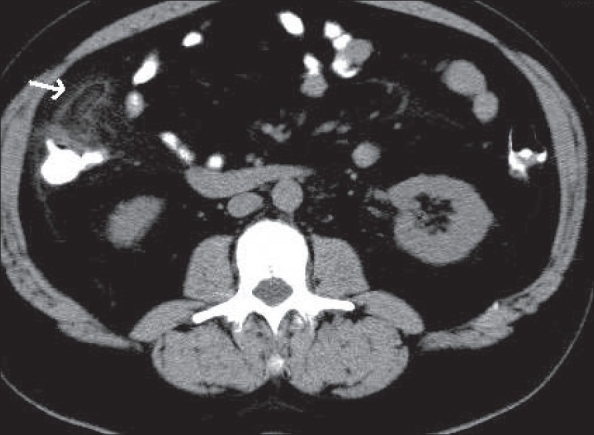
Epiploic appendagitis. Contrast-enhanced CT scan shows a fat density lesion (arrow) with a hyperdense rim and fat stranding adjacent to the ascending colon

It is a benign self-limiting condition due to spontaneous torsion, inflammation, or venous thrombosis of the draining vein of one of the epiploic appendages of the colon. CT demonstrates a pericolonic lesion with fat-attenuation, with a well-defined hyperattenuating rim and associated mild periappendageal fat stranding[15] [Figure 14]. Sometimes there may be focal thickening of the adjacent colon and mild thickening of the adjacent parietal peritoneum. Typical CT features of epiploic appendage inflammation and a normal or nonvisualized appendix suggest this diagnosis.

### 7. Right-sided diverticulitis

**Figure 15(A, B):**
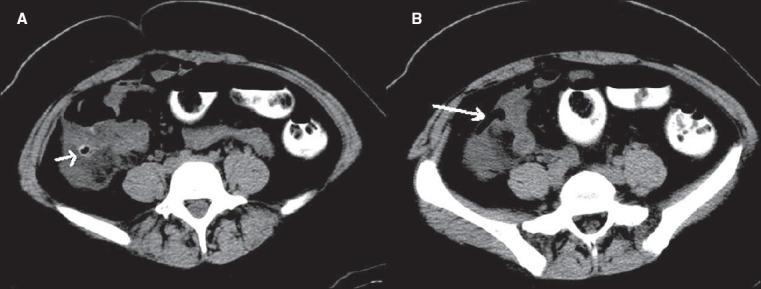
Cecal diverticulitis. Plain CT scan with oral contrast shows an inflamed right-sided cecal diverticulum (short arrow in A) with surrounding fat stranding and an air-filled normal appendix (long arrow in B)

Diverticular disease is more common in the Western population, 95% involving the left colon and 1-5%, the right.[16] The presence of colonic diverticuli, focal colonic wall thickening, and pericolonic inflammation in the setting of a normal appendix, suggest this diagnosis[17] [Figure 15]. Complications of diverticulitis include pericolonic abscess, phlegmon formation, and perforation.

**Figure 16:**
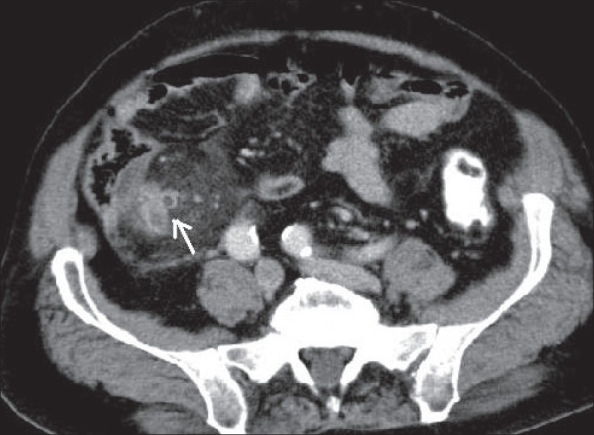
Perforated ileal diverticulitis. Contrast-enhanced CT scan shows multiple ileal diverticuli with an inflamed diverticulum (arrow) in the terminal ileum with adjacent inflammation and a few extraluminal air loculi, consistent with perforation

**Figure 17:**
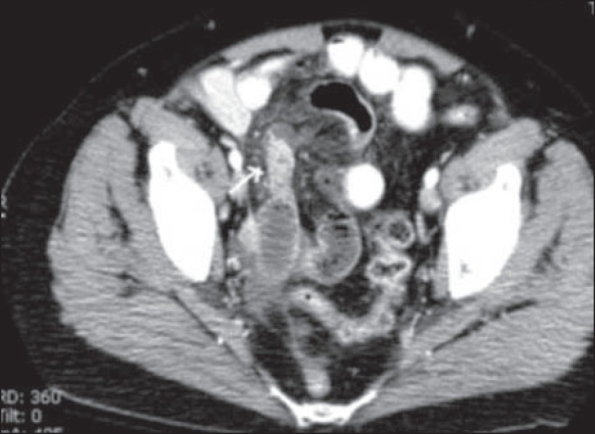
Meckel's diverticulitis: Contrast-enhanced CT scan shows a dilated tubular structure (arrow), arising from the terminal ileum with surrounding inflammatory changes in the mesentery

Small bowel diverticulitis is uncommon, but ileal diverticulitis can also clinically mimic appendicitis [Figure 16] as can Meckel's diverticulitis. Meckel's diverticulum arises from the antimesenteric border of the small bowel as a blind-ending tubular structure, containing fluid, air, or particulate material; it may be located in the right lower quadrant or near the midline.[18] CT findings include an inflamed diverticulum with mural enhancement, thickening, and associated inflammatory changes in the mesentery [Figure 17] (the epicenter being more towards the midline) in the setting of a normal appendix.

### 8. Right-sided colitis

**Figure 18(A, B):**
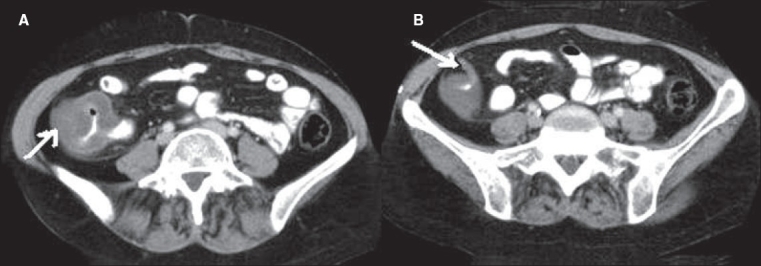
Colitis. Contrast-enhanced CT scan shows circumferential wall thickening of the ascending colon and cecum with surrounding fat stranding (arrow in Figure A). The terminal ileum and appendix (arrow in Figure B) are normal

Inflammatory and infective conditions may involve the right colon and simulate appendicitis. However, the extent of colonic wall thickening is much greater than with appendicitis. CT findings include circumferential wall thickening of the colon with adjacent pericolonic fat stranding [Figure 18]. Neutropenic typhlitis (ileocecal syndrome or neutropenic colitis) is seen in neutropenic patients or patients on immunosuppressive therapy[19] and presents on CT with ileocecal wall thickening, pericolonic fat stranding, and pericolonic fluid collections, along with pneumatosis coli and intramural abscesses in more advanced cases.

### 9. Cecal volvulus

**Figure 19(A, B):**
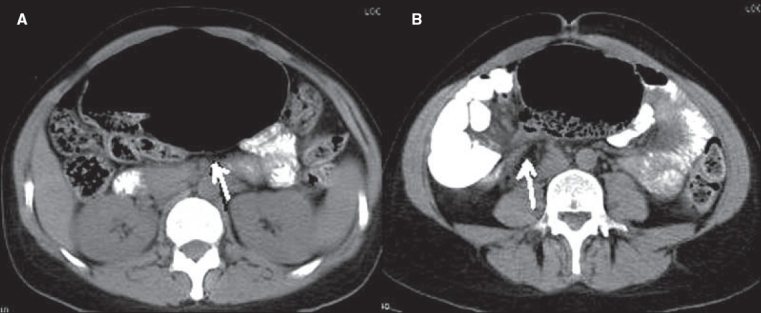
Cecal volvulus. Plain CT study shows a dilated cecum (arrow in A) with an air-fluid level reaching up to the left upper abdomen, demonstrating a beak sign (arrow in B). The appendix is not definitely visualized

Cecal volvulus accounts for 10% of all large bowel obstruction. It commonly occurs in patients with incomplete right colon fixation, which leads to excessive cecal mobility and the potential for vascular compromise. CT findings include an abnormally dilated cecum, which is comma or bean-shaped, located most frequently to the left of the abdomen. There is associated small bowel obstruction in 50% of cases. The ‘whirl sign’ (whirled configuration of the fatty mesentery and mesenteric vessels at the site of torsion)[20] and ‘beak sign’ (converging point of afferent and efferent loops of the dilated cecum at the point of torsion, resembling a bird's beak) help diagnose cecal volvulus [Figure 19]. The volvulus may sometimes be associated with signs of vascular compromise, which includes mural thickening, pneumatosis coli, and mesenteric fat stranding.

### 10. Bowel intussusception

**Figure 20:**
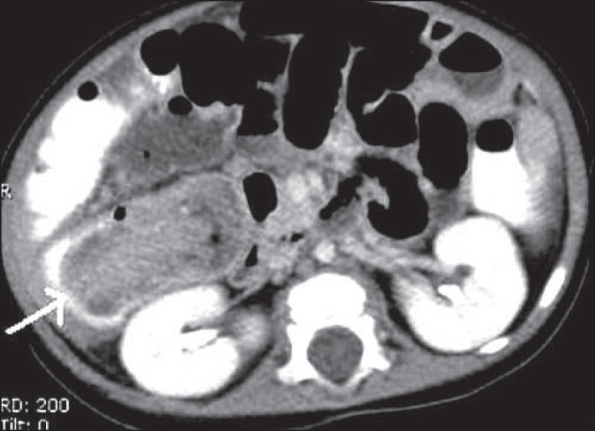
Ileocolic intussusception. Contrast-enhanced CT scan shows a ‘bowel in bowel’ configuration (arrow) with mesenteric fat invagination in the right lower quadrant

Intussusception is a condition in which a portion of the bowel invaginates into an adjacent segment of bowel. Intussusception is primarily a disease of infants and children and only about 5% of cases occur in adults.[21] On CT, intussusception appears as an abnormal target-like mass and may be associated with small bowel obstruction. The lead point may or may not be seen. The presence of a ‘bowel-in-bowel’ configuration, with or without mesenteric fat or vessels, is a diagnostic CT finding [Figure 20].

### 11. Right ovarian vein thrombosis

**Figure 21:**
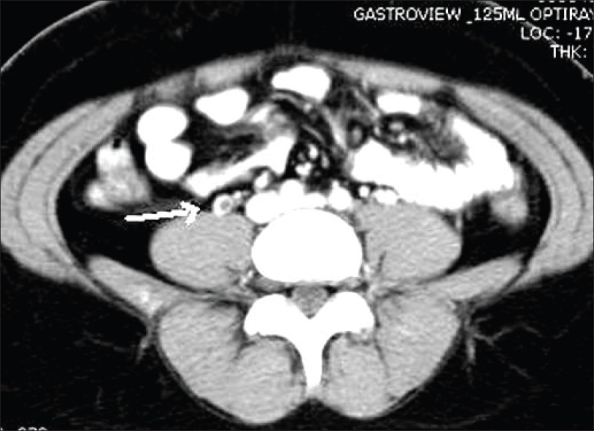
Ovarian vein thrombosis. Contrast-enhanced CT scan shows a filling defect in the right ovarian vein (arrow)

Ovarian vein thrombosis is an uncommon disorder, usually associated with pelvic conditions such as recent childbirth, pelvic inflammatory disease, malignancies, and pelvic surgery. The right ovarian vein is involved in almost 90% of cases.[22] On contrast-enhanced CT, the ovarian vein is enlarged and demonstrates a central hypodensity which extends from the level of the pelvis to the infrarenal inferior vena cava, with associated perivascular fat stranding [Figure 21].

### 12. Ovarian mass/cyst

**Figure 22:**
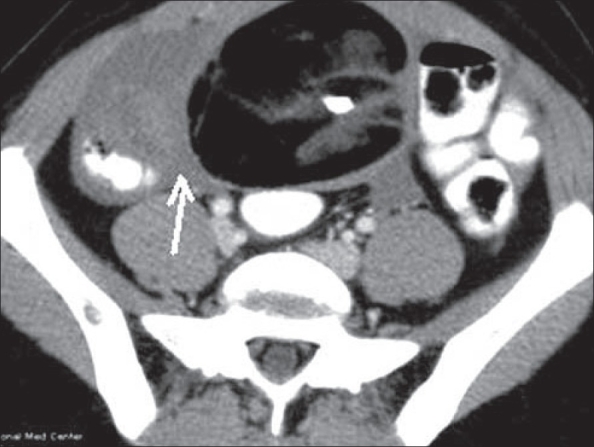
Ovarian dermoid with torsion. Contrast-enhanced CT scan shows a large right ovarian dermoid with high-density peritoneal fluid due to hemorrhage (arrow), strongly suggesting the presence of associated torsion. There were signs of torsion on Doppler USG as well. The appendix was normal

Complications of ovarian masses and cysts include rupture, torsion, or hemorrhage, all of which usually present as acute lower quadrant pain. On CT, the presence of an ovarian mass with areas of fat attenuation, calcification, teeth, or fat-fluid levels confirms the diagnosis of dermoid[23] [Figure 22]. Ectopic pregnancy may present as right lower abdominopelvic pain. CT findings include presence of an adnexal mass, enlarged uterus, and hemorrhagic free fluid (in case of rupture). However, this diagnosis is most accurately made on USG.

### 13. Bowel ischemia

**Figure 23(A, B):**
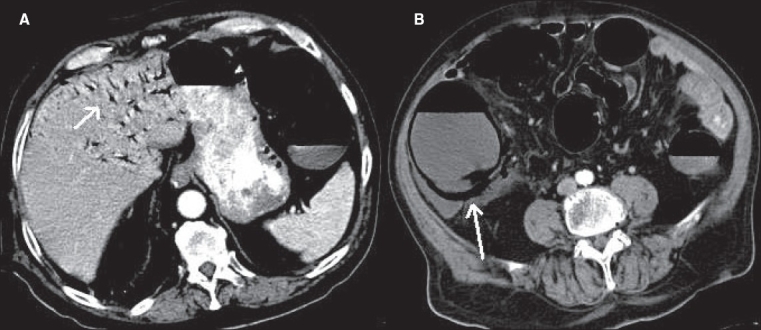
Bowel infarction with perforation. Contrast-enhanced CT shows portal venous air (short arrow in A) and bowel pneumatosis (long arrow in B) with dilated bowel loops

Bowel ischemia is a common cause of acute abdomen in the elderly population. CT findings include bowel wall thickening due to edema or hemorrhage, with lack of enhancement, along with portal venous gas and pneumatosis coli, which indicate infarction [Figure 23]. Pneumoperitoneum may be seen in cases of perforation. Contrast-enhanced CT may demonstrate a thrombus in the involved vessel.

## Summary

Acute appendicitis is the most common cause of right lower quadrant pain, but other diagnoses should also be considered. Spiral CT is an extremely useful imaging modality for the investigation of right lower quadrant pain.
